# Transcriptome Analysis in *Haematococcus pluvialis*: Astaxanthin Induction by High Light with Acetate and Fe^2+^

**DOI:** 10.3390/ijms19010175

**Published:** 2018-01-07

**Authors:** Bangxiang He, Lulu Hou, Manman Dong, Jiawei Shi, Xiaoyun Huang, Yating Ding, Xiaomei Cong, Feng Zhang, Xuecheng Zhang, Xiaonan Zang

**Affiliations:** Key Laboratory of Marine Genetics and Breeding, Ministry of Education, Ocean University of China (OUC), Qingdao 266003, Shandong, China; hbxwork@163.com (B.H.); hoululu@ouc.edu.cn (L.H.); dongmanman55@163.com (M.D.); shijiawei@stu.ouc.edu.cn (J.S.); hxy764379760@163.com (X.H.); 17864272859@163.com (Y.D.); 13371370369@163.com (X.C.); zfhhxxttxs@163.com (F.Z.); xczhang8@163.com (X.Z.)

**Keywords:** *Haematococcus pluvialis*, astaxanthin, high light stress, salt stress, transcriptome

## Abstract

*Haematococcus pluvialis* is a commercial microalga, that produces abundant levels of astaxanthin under stress conditions. Acetate and Fe^2+^ are reported to be important for astaxanthin accumulation in *H. pluvialis*. In order to study the synergistic effects of high light stress and these two factors, we obtained transcriptomes for four groups: high light irradiation (HL), addition of 25 mM acetate under high light (HA), addition of 20 μM Fe^2+^ under high light (HF) and normal green growing cells (HG). Among the total clean reads of the four groups, 156,992 unigenes were found, of which 48.88% were annotated in at least one database (Nr, Nt, Pfam, KOG/COG, SwissProt, KEGG, GO). The statistics for DEGs (differentially expressed genes) showed that there were more than 10 thousand DEGs caused by high light and 1800–1900 DEGs caused by acetate or Fe^2+^. The results of DEG analysis by GO and KEGG enrichments showed that, under the high light condition, the expression of genes related to many pathways had changed, such as the pathway for carotenoid biosynthesis, fatty acid elongation, photosynthesis-antenna proteins, carbon fixation in photosynthetic organisms and so on. Addition of acetate under high light significantly promoted the expression of key genes related to the pathways for carotenoid biosynthesis and fatty acid elongation. Furthermore, acetate could obviously inhibit the expression of genes related to the pathway for photosynthesis-antenna proteins. For addition of Fe^2+^, the genes related to photosynthesis-antenna proteins were promoted significantly and there was no obvious change in the gene expressions related to carotenoid and fatty acid synthesis.

## 1. Introduction

*Haematococcus pluvialis* is a unicellular freshwater microalga that is rich in astaxanthin. The red ketocarotenoid astaxanthin (3,30-dihydroxy-β,β-carotene-4,40-dione) is the most powerful biological antioxidant and is highly demanded in health care and aquaculture, with the latest research showing that astaxanthin may play roles in anti-depression and preventing UVA-induced skin photoaging [1,2,3,4]. Astaxanthin is a 40-carbon isoprenoid and is synthesized from isopentenyl diphosphate (IPP) and dimethylallyl diphosphate (DMAPP). Three IPP molecules are added to DMAPP sequentially to form 20-carbon geranylgeranyl pyrophosphate (GGPP) and two GGPP molecules bind head-to-tail to form a tetraterpene phytoene. The phytoene is transformed to lycopene by dehydrogenation and then to β-carotene by cyclization. Finally, astaxanthin is generated from β-carotene [5,6,7]. When the cells of *H. pluvialis* are under stress conditions, such as nutrient deprivation, high irradiation, or high salt concentration, astaxanthin is produced in the form of fatty acyl mono-or diesters [7].

*H. pluvialis* is now the best source of natural astaxanthin, constituting up to 4% of cellular dry weight [8,9,10]. The life cycle of *H. pluvialis* can be divided into four stages: I, vegetative cell growth; II, encystment (vegetative to immature cyst cells); III, maturation (immature to mature cyst cells); and IV, germination (mature cyst to vegetative cells) [11]. Astaxanthin is accumulated mainly in stage II and stage III. Many studies attempting to increase astaxanthin accumulation in *H. pluvialis* by optimization of induction conditions have been reported. Jeon found that 30 mM acetate and 170 μEm^−2^·s^−1^ light intensity was good for astaxanthin accumulation in *H. pluvialis* [12]. Kobayashi pointed out that algal astaxanthin formation was more enhanced by the addition of acetate plus Fe^2+^ than by the addition of acetate alone in *H. pluvialis*, from which he concluded that oxidative stress was involved in the posttranslational activation of carotenoid biosynthesis in acetate-induced cyst cells [13]. Hong successfully used 50 μM Fe^2+^ to enhance the production of astaxanthin [14]. Following-up such studies, we chose light, acetate and Fe^2+^ as variables to explore the molecular mechanism of astaxanthin production in the red-cell stage of *H. pluvialis* by transcriptome analysis.

Moreover, it has been reported that microalgae can produce fatty acids with unusual chain lengths and a high degree of unsaturation, which are not found in significant amounts in nature elsewhere. Prominent examples are the polyunsaturated fatty acids (PUFAs) eicosapentaenoic acid (EPA, 20:5) or docosahexaenoic acid (DHA, 22:6), ω-3 fatty acids, that are commercially produced as human health supplements [15,16]. In *H. pluvialis*, the accumulation of astaxanthin is correlated with increasing fatty acid biosynthesis under stress conditions [17]. Gwak indicated that astaxanthin mono- and diesters form lipid globules with triacylglycerol (TAG) based on an analysis of lipidomes and transcriptomes of *H. pluvialis* cultured under high irradiance [7]. In *H. pluvialis*, the lipid content is close to 35% of the dry weight under stress conditions and the content of polyunsaturated fatty acids (PUFAs) is closed to 50% of the neutral lipid [18]. This suggests that *H. pluvialis* could also produce fatty acids as a byproduct which could be used in human health supplements.

RNA sequencing (RNA-seq) is a newly developed deep-sequencing technology for transcriptome analysis, which has been a key technique for biological inquiry for decades [19]. In the present study, the transcriptomes of *H. pluvialis* were sequenced using Illumina Hiseq2000 (Illumina, Santiago, CA, USA). Through transcriptome analysis of *H. pluvialis*, we attempt to find the specific changes in metabolic pathways of algal cells grown with acetate and Fe^2+^ under high light, which may lay a foundation for insight into the astaxanthin synthesis mechanism in *H. pluvialis* and also provide guidance for production of astaxanthin in industrial farming.

## 2. Result

### 2.1. Short-Read De Novo Sequencing and Assembly

**Figure 1:**
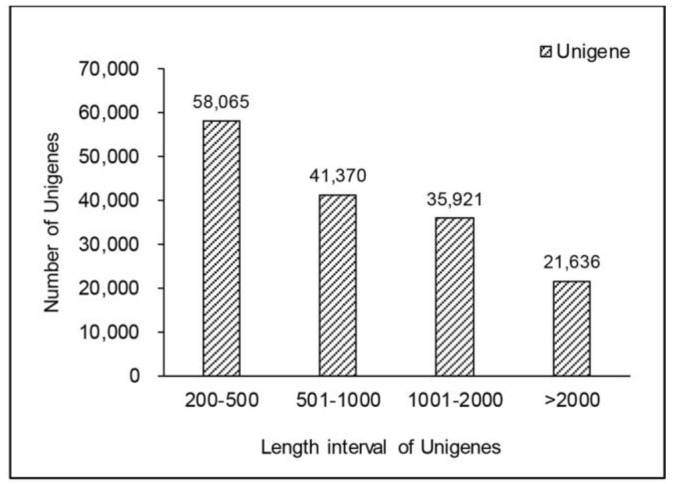
The length distribution of unigenes.

By sequencing the transcriptome of *H. pluvialis*, fifty to sixty-five million raw reads of each sample were obtained. To guarantee the quality of data used for the analyses, stringent parameters were used to measure the quality of reads and all reads with undetermined information greater than 10% were removed. After this filtering, about sixty million clean reads of each sample were generated, with more than 95% bases showing quality greater than Q20, while the GC percentage was around 60% (Table 1). Then, the k-mer (*k* = 25) technique was used to measure the nucleotide sequence of reads. As a no-reference genome project, these clean reads were assembled into contigs using overlap information until the contigs could not be further extended. After a series of subsequent steps, 156,992 unigenes (>200 bp) were generated, with an N50 of 1620 bp and N90 of 456 bp. The size of the unigenes varied from 201 bp to 35,138 bp with a mean length of 1047 bp and a total length of 164,437,994 bp (Figure 1).

### 2.2. Function Annotation

To obtain comprehensive information on the function of *H. pluvialis* genes, all of the unigenes were blasted against seven databases, including: NR (NCBI non-redundant protein sequences), NT (NCBI nucleotide sequences), KO (KEGG Ortholog), SwissProt (A manually annotated and reviewed protein sequence database), PFAM (Protein family), KOG (Clusters of Orthologous Groups of proteins), and GO (Gene Ontology). As a whole, there were 76,744 unigenes (48.88%) annotated in at least one database, 5918 unigenes (3.76%) were annotated in all of the databases. Specifically, 55,835 unigenes (35.56%), 60,846 unigenes (38.75%), 58,643 unigenes (37.35%), 23,632 unigenes (15.05%) and 21,569 unigenes (13.73%) were annotated in NR, GO, PFAM, KO and KOG, respectively (Table 2). 

**Figure 2:**
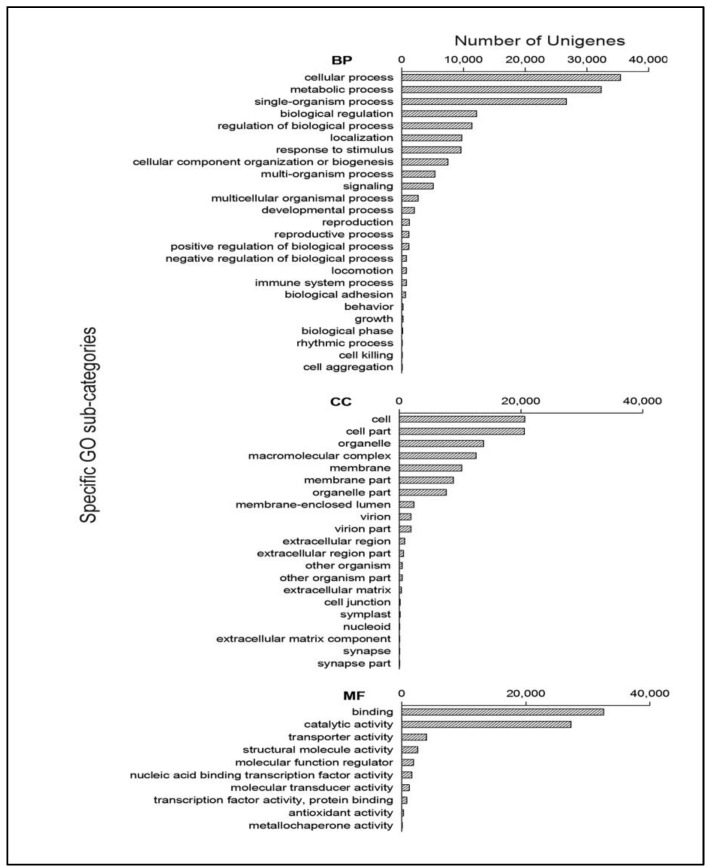
Gene function classification of all unigenes annotated by GO. “BP” represents “Biological Process” domain, “CC” represents “Cellular Component” domain and “MF” represents “Molecular Function” domain.

After the GO annotation, the annotated unigenes were classified according to the three GO categories: Biological Process (BP), Cellular Component (CC) and Molecular Function (MF). All of the 60,846 unigenes annotated in the GO database were classified into 56 sub-categories. The top three sub-categories for BP were cellular process, metabolic process and single-organism process, for which the numbers of genes were 35,416 (58.21% of the 60,846 unigenes annotated to GO), 32,315 (53.11%) and 26,630 (43.77%) respectively (some of the unigenes could annotated in more than one category). The top three sub-categories of CC were cell part, cell and organelle, for which the numbers of genes were 20,557 (33.79%), 20,577 (33.79%) and 7693 (12.64%) respectively. The top three sub-categories of MF were binding, catalytic activity and transporter activity, for which the numbers of genes were 32,522 (53.45%), 27,253 (44.79%) and 3974 (6.53%) respectively (Figure 2).

**Figure 3:**
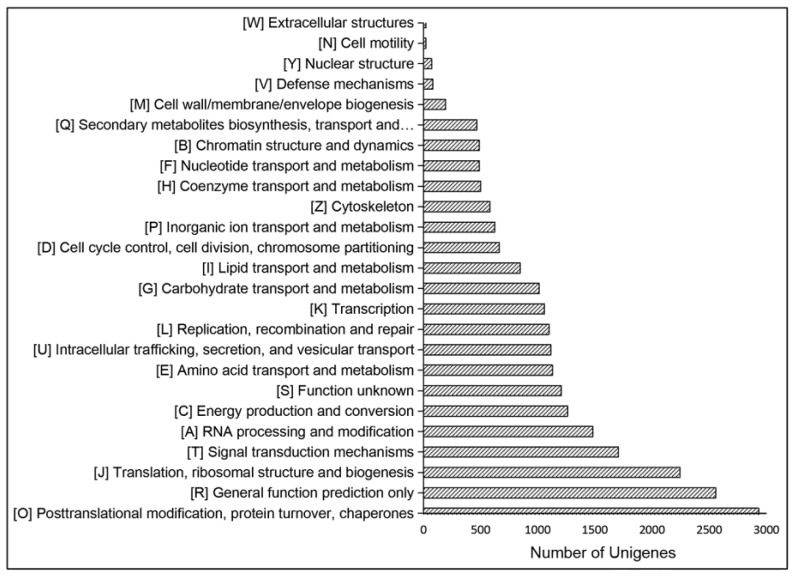
KOG functional classification of the unigenes. KOG: Clusters of Orthologous Groups of proteins database.

According to the annotations in the KOG database, 21,569 annotated unigenes were subdivided to 25 groups. The top KOG category was “Posttranslational modification, protein turnover, chaperones” (2931, 13.58%), followed by “General function prediction only” (2557, 11.85%). The smallest groups were “Cell motility” (21, 0.09%) and “Extracellular structures” (21, 0.09%) (Figure 3).

**Figure 4:**
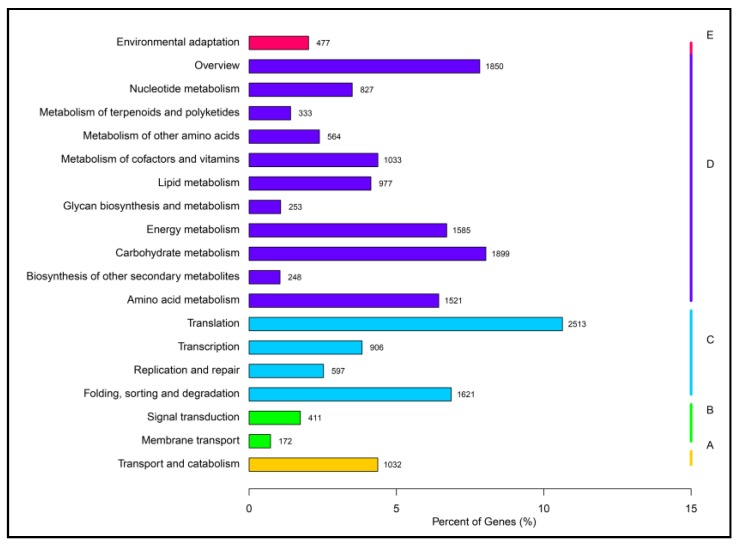
KEGG Classification. KEGG means Kyoto Encyclopedia of Genes and Genomes. The *Y* axis gives specific pathways in the second hierarchy. “A, B, C, D and E” (yellow, green, blue, purple and pink) represent five groups of pathways in the first hierarchy: Cellular Processes, Environmental Information Processing, Genetic Information Processing and Organismal Systems.

The 23,632 unigenes annotated using the KO database were classified into five pathways of “hierarchy 1.” These are Cellular Processes, Environmental Information Processing, Genetic Information Processing and Organismal Systems. As for the number of unigenes in “hierarchy 2,” the largest number was 2513 unigenes (10.63%) in the pathway for “Translation,” followed by 1899 unigenes (8.03%) in the pathway for “Carbohydrate metabolism” (Figure 4).

### 2.3. Analysis of DEGs

#### 2.3.1. DEG Profiles in Response to the Treatments

**Figure 5:**
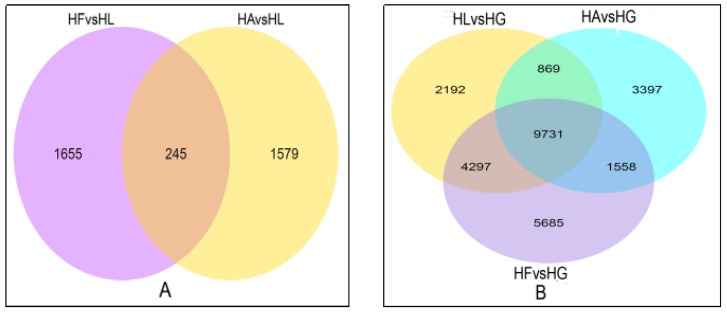
Venn Diagram of DEGs (differentially expressed genes). (**A**) was the comparison of DEGs in HAvsHL and HFvsHL; (**B**) was the comparison of DEGs in HLvsHG, HAvsHG and HFvsHG.

**Figure 6:**
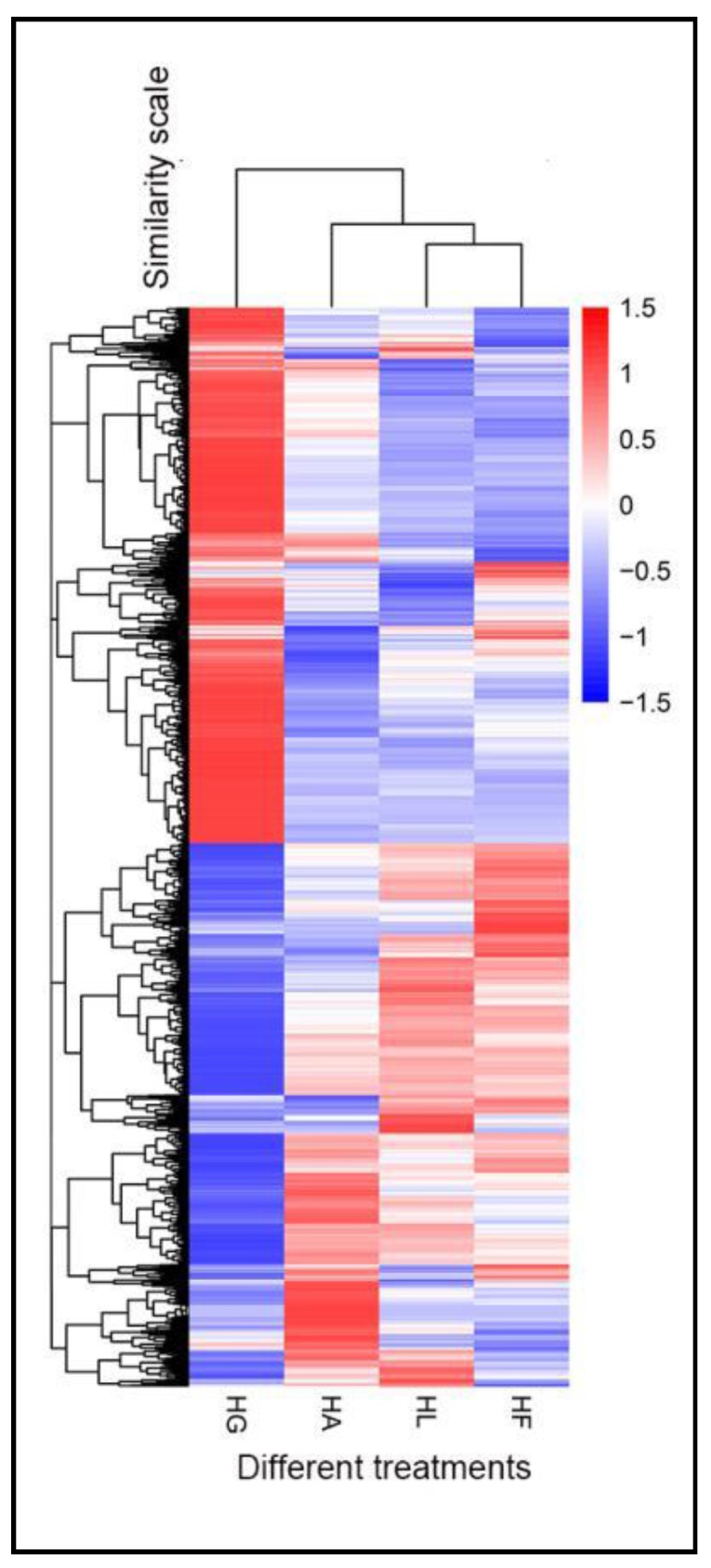
Cluster analysis of DEGs. The *Y* axis represents relative expression, the red and blue represent up-regulation and down-regulation. HF: the group of addition of Fe^2+^ together with high light treatment; HL: high light treatment group; HA: the group of addition of acetate together with high light treatment; HG: green growth group.

To investigate the effect of a different inducing factor of *H. pluvialis*, six comparison groups of DEGs were set as follows: HLvsHG—high light treatment group (HL) compared with green growth group (HG); HAvsHG—addition of acetate together with high light treatment (HA) compared with green growth group; HAvsHL—addition of acetate together with high light treatment compared to only high light treatment; HFvsHG—addition of Fe^2+^ together with high light treatment (HF) compared with green growth group; HFvsHL—addition of Fe^2+^ together with high light treatment compared to only high light treatment; HAvsHF—acetate treatment compared with Fe^2+^ treatment. The DEG numbers for each comparison group are shown in Table 3. When using HG as control, there were more DEGs in HAvsHG, HFvsHG and HLvsHG groups than using HL as control, like HAvsHL and HFvsHL groups. The Venn Diagram of DEGs (Figure 5) showed that there were 9731 similar DEGs compared to HG group and 245 similar DEGs compared to HL group. Cluster analysis of DEGs was used for judging the expression pattern of DEGs under different experimental conditions (Figure 6). The cluster of DEGs showed that HL, HF and HA had significantly different expression patterns than HG and HF was more similar to HL, which means that stress, high light, acetate and Fe^2+^, caused a pronounced change in gene expression and that the effect of acetate was greater than that of Fe^2+^.

#### 2.3.2. DEG Analysis in the High Light Treatment

There were 17,089 DEGs composed of 8243 up-regulated genes and 8846 down-regulated genes in the pairwise comparison of HLvsHG. To generate an overview of the high-light-responsive DEGs, a GO analysis was performed. The GO enrichment analysis revealed that 46 GO terms were significantly enriched (corrected *p* value < 0.05) in the DEGs responding to high light irradiation. The main categories were in the “Biological Process” domain, including “metabolic process,” “single-organism process” and “single-organism cellular process.” The main categories in the “Molecular Function” domain were “hydrolase activity, acting on glycosyl bonds,” “hydrolase activity, hydrolyzing O-glycosyl compounds” and “phosphorus-oxygen lyase activity.” There was only one category in the “Cellular Component” domain, which was “ribonucleotide-diphosphate reductase complex” (Table S1).

To identify the biological pathways that were active under high light irradiation, the up-regulated and down-regulated DEGs were mapped to canonical signaling pathways in the KEGG database. The 8243 up-regulated DEGs were significantly enriched (*p* < 0.05) in 22 KEGG terms. Among these, numerous genes were involved in nucleotide metabolism, such as “Amino sugar and nucleotide sugar metabolism,” “DNA replication,” “Purine metabolism,” “Pyrimidine metabolism.” Another large DEG group was enriched in lipid-related pathways, such as “Fatty acid elongation,” “Fatty acid degradation,” “Folate biosynthesis,” “Ascorbate and aldarate metabolism,” “alpha-Linolenic acid metabolism,” “One carbon pool by folate.” The 8846 down-regulated DEGs were significantly enriched in 19 KEGG terms, which were related to metabolism (“Purine metabolism,” “Pyruvate metabolism,” “Propanoate metabolism,” “Glycerolipid metabolism,” “Taurine and hypotaurine metabolism”) and biosynthesis (“Carotenoid biosynthesis,” “Fatty acid biosynthesis”). In summary, the genes related to fatty acid pathways and the carotenoid pathway are changed under high light condition. 

#### 2.3.3. DEG Analysis for Addition of Acetate under High Light Condition

There were 15,555 (7699 up-regulated and 7856 down-regulated) DEGs in the comparison of HAvsHG (acetate treatment group under high light condition compared with green growth group) and 1824 (1121 up-regulated and 703 down-regulated) DEGs in the comparison of HAvsHL (acetate treatment group compared with only high light irradiation group). GO enrichment analysis showed that 78 GO terms were significantly enriched in the comparison of HAvsHG. The main categories were in “Biological Process” and “Molecular Function” domain, including “single-organism process,” “single-organism cellular process,” “single-organism cellular process” and “catalytic activity.” There were also some categories related to fatty acid, such as “fatty acid biosynthetic process” and “fatty acid metabolic process.” There were only five significant terms in the “Cellular Component” domain, these were viral envelope, viral membrane, exodeoxyribonuclease VII complex, obsolete flagellum, nematocyst. The top 3 significantly enriched terms were “cyclic nucleotide biosynthetic process,” “cyclic nucleotide metabolic process” (Biological Process) and “phosphorus-oxygen lyase activity” (Molecular Function) (Table S2). Further analysis of DEGs in HAvsHL could lead us to directly understand the function of acetate by subtracting the expression effects due to the common high light factor. The 1824 DEGs were significantly enriched to 139 GO terms, in which the main categories were also in the “Biological Process” and “Molecular Function” domains, including “metabolic process,” “catalytic activity,” “single-organism process,” “single-organism metabolic process,” “oxidoreductase activity” and “oxidation-reduction process.” Besides the categories related to fatty acid (“fatty acid biosynthetic process,” “fatty acid metabolic process”), there were some categories related to oxidation-reduction processes (“oxidoreductase activity, acting on the CH-CH group of donors,” “oxidoreductase activity, acting on CH-OH group of donors,” etc.) and response processes (“response to abiotic stimulus,” “response to light intensity,” etc.). Moreover, there were 19 terms significantly enriched in the “Cellular Component” domain, most of which were related to photosynthesis components, such as “chloroplast,” “thylakoid part,” “photosynthetic membrane,” “chloroplast stroma,” etc. (Table S3). 

Using the KEGG database to analyze the DEGs in the HAvsHL comparison, the up-regulated genes were significantly enriched in 18 KEGG terms, including “Carbon fixation in photosynthetic organisms,” “Fatty acid elongation,” “Taurine and hypotaurine metabolism,” “Biosynthesis of unsaturated fatty acids,” “Carotenoid biosynthesis,” “Linoleic acid metabolism” and so on. The down-regulated genes were significantly enriched in 11 KEGG terms, including “DNA replication,” “Amino sugar and nucleotide sugar metabolism,” “Oxidative phosphorylation,” “Photosynthesis,” “Photosynthesis-antenna proteins” and so on.

#### 2.3.4. DEG Analysis for Addition of Fe^2+^ under High Light Condition

There were 21,271 (10,496 up-regulated and 10,775 down-regulated) DEGs in the HFvsHG comparison and 1900 (1182 up-regulated and 718 down-regulated) DEGs in HFvsHL comparison. The 21,271 DEGs in HFvsHG were significantly enriched in 43 GO terms, in which the main categories were in “Biological Process” and “Molecular Function” domain, including “metabolic process,” “catalytic activity,” “organic substance metabolic process,” “single-organism process,” “primary metabolic process” and no categories were in the “Cellular Component” domain. The top 5 significantly enriched categories were “cyclic nucleotide biosynthetic process,” “nucleoside phosphate biosynthetic process,” “nucleotide biosynthetic process,” “cyclic nucleotide metabolic process” (Biological Process) and “phosphorus-oxygen lyase activity” (Molecular Function) (Table S4). The 1900 DEGs in HFvsHL comparison were significantly enriched in 57 GO terms, in which the main categories were in the “Biological Process” and “Cellular Component” domains, including “metabolic process,” “cellular metabolic process,” “cell” and “cell part.” There were only five categories in the “Molecular Function” domain, including “acireductone dioxygenase [iron(II)-requiring] activity,” “beta-glucosidase activity,” “nutrient reservoir activity,” “structural constituent of ribosome,” “structural molecule activity.” The top 5 significantly enriched categories were “thylakoid,” “photosynthetic membrane,” “thylakoid part,” “chloroplast” (Cellular Component) and “photosynthesis” (Biological Process), which were related to photosynthesis (Table S5).

Using the KEGG database to analyze the DEGs in the HFvsHL comparison, the up-regulated genes were significantly enriched in 6 KEGG terms, including “Photosynthesis,” “Photosynthesis-antenna proteins,” “Ribosome,” “Glyoxylate and dicarboxylate metabolism,” “Carbon fixation in photosynthetic organisms” and “Flavonoid biosynthesis.” The down-regulated genes were significantly enriched in 5 KEGG terms, including “Ribosome,” “Oxidative phosphorylation,” “Tryptophan metabolism,” “Fatty acid elongation,” “Porphyrin and chlorophyll metabolism”.

#### 2.3.5. KEGG Analysis of DEGs between Two Groups: Addition of Acetate or Fe^2+^

The above DEG analyses indicated that the two salts had different influences on *H. pluvialis* under high light stress. The 4864 (2844 up-regulated and 2020 down-regulated) DEGs in the HAvsHF comparison were analyzed using the KEGG database. The 2844 up-regulated DEGs were significantly enriched in 17 KEGG terms, such as “Fatty acid elongation,” “Fatty acid biosynthesis,” “Linoleic acid metabolism,” “Glycerolipid metabolism,” “Biosynthesis of unsaturated fatty acids,” which related to lipid substances and “Carotenoid biosynthesis,” which related to astaxanthin synthesis. The 2020 down-regulated DEGs were significantly enriched in 12 KEGG terms, in which the main terms were related to DNA replication (“DNA replication,” “Mismatch repair,” “Pyrimidine metabolism,” “Porphyrin and chlorophyll metabolism”) and photosynthesis (“Photosynthesis,” “Photosynthesis-antenna proteins,” “Carbon fixation in photosynthetic organisms,” “Porphyrin and chlorophyll metabolism”). All the above shows that acetate promotes the up-regulated expression of genes related to lipid and carotenoid pathways in *H. pluvialis*. As for Fe^2+^, the expression of genes that related to photosynthesis were significantly promoted compared with the inhibition effect of sodium acetate.

### 2.4. The Effects of Induction Treatment on Specific Biological Pathways

In order to explore the specific effects of the three induction treatments (high light, acetate, Fe^2+^) on *H. pluvialis*, based on the DEG analysis in KEGG, three specific pathways were chosen: “carotenoid biosynthesis,” “fatty acid biosynthesis” and “photosynthesis-antenna proteins”.

#### 2.4.1. The Effects on the Carotenoid Biosynthesis Pathway

**Figure 7:**
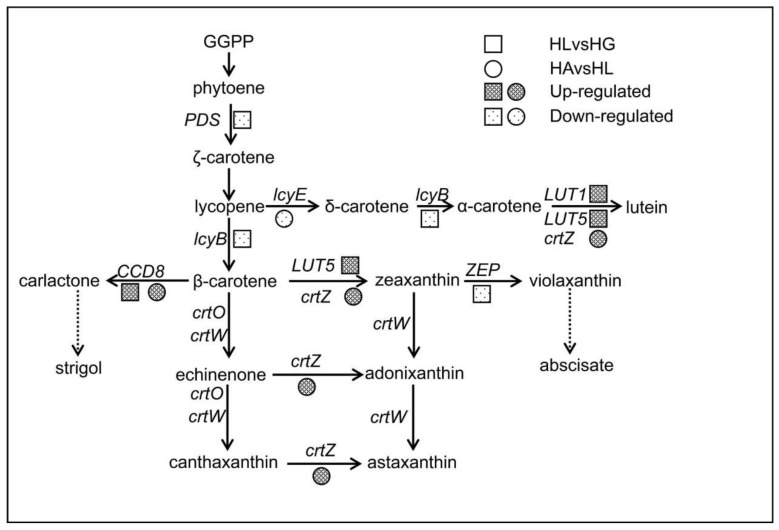
The pathways involved in carotenoid biosynthesis. The “solid arrow” means the related genes are showed in figure and the “dotted arrow” means the genes associated with the pathway are omitted. GGPP: geranylgeranyl pyrophosphate; PDS: phytoene desaturase; ZEP: zeaxanthin epoxidase; CCD8: carotenoid cleavage dioxygenase.

In HLvsHG, the 22 DEGs related to carotenoid biosynthesis (pathway map: ko00906) were annotated as 8 genes: phytoene desaturase (*PDS*), lycopene β-cyclase (*lcyB*), prolycopene isomerase (*crtISO*), carotene epsilon-monooxygenase (*LUT1*), zeaxanthin epoxidase (*ZEP*), beta-carotene hydroxylase (*crtZ*), β-ring hydroxylase (*LUT5*), carotenoid cleavage dioxygenase 8 (*CCD8*). In HAvsHL, the 7 DEGs related to carotenoid biosynthesis were annotated as 3 genes: *CCD8*, *crtZ* and lycopene epsilon-cyclase (*lcyE*) and there were no DEGs in HFvsHL (Table 4). *CrtZ* and *LUT5* are genes related to the conversion of β-carotene to astaxanthin and in addition, they also play a role in the conversion of lycopene to lutein with *LUT1* (Figure 7). The expression of *crtZ* was increased under high light and significantly increased in the acetate treatment. *LUT5* and *LUT1* were up-regulated under high light but did not change in the comparison groups of HAvsHL and HFvsHL. The genes in the conversion of geranylgeranyl pyrophosphate (GGPP) to β-carotene, including *PDS*, *crtISO*, *lcyB*, were down-regulated under high light in this study. The results indicate that not all the genes involved in astaxanthin synthesis were up-regulated under the stress condition, maybe due to the stress duration, stress condition or other reasons. *LcyE* is an important gene in the lycopene to lutein synthesis pathway and it was down-regulated in HAvsHL, which would decrease competition for the precursor of β-carotene. *ZEP* converts zeaxanthin to violaxanthin under low-light condition, which would further generate abscisate. *ZEP* was down-regulated in HLvsHG, which means that the conversion of zeaxanthin to violaxanthin was strongly inhibited under high light. The last gene *CCD8* is related to the conversion of β-carotene to strigol (Figure 7). *CCD8* was significantly up-regulated both in HLvsHG and HAvsHL, which indicates that strigol production is enhanced, together with astaxanthin, for both the high light and the high light with acetate treatments. No significantly enriched DEGs were related to carotenoid biosynthesis in HFvsHL, which indicates that addition of Fe^2+^ probably did not influence the accumulation of astaxanthin.

#### 2.4.2. The Effects on the Fatty Acid Elongation Pathway

**Figure 8:**
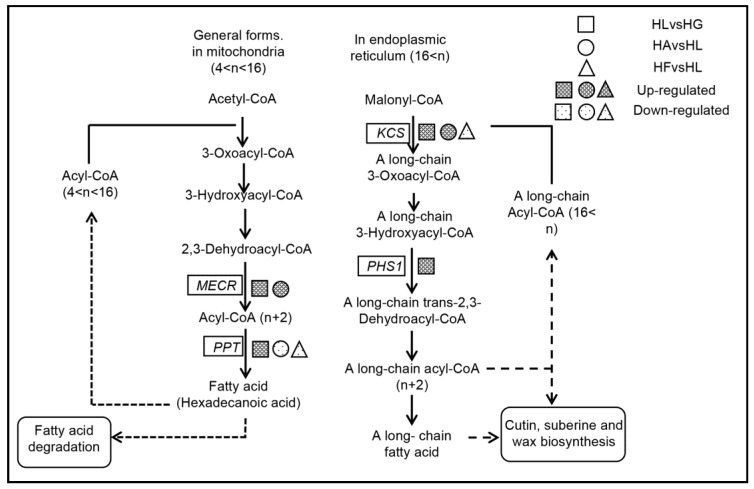
The pathways for fatty acid elongation. The boxed genes are the differentially expressed genes related to the pathway, “*n*” indicates the number of carbon atoms in the fatty acid chain. *MECR*: mitochondrial trans-2-enoyl-CoA reductase; *PPT*: palmitoyl-protein thioesterase; *KCS*: 3-ketoacyl-CoA synthase; *PHS1*: very-long-chain (3R)-3-hydroxyacyl-CoA dehydratase.

Fatty acid elongation (pathway map: ko00062) can be divided into two pathways, one to form palmitic acid in mitochondria, (0 < *n* < 16, where “*n*” is the number of carbon atoms in the fatty acid chain) and the second to form long-chain fatty acids (*n* > 16) in the endoplasmic reticulum (Figure 8). There were 28 differentially expressed unigenes related to the fatty acid elongation pathways in the HLvsHG comparison group and all of them were up-regulated. Among those, 25 unigenes annotated as 3-ketoacyl-CoA synthase (*KCS*) and the other three were mitochondrial *trans*-2-enoyl-CoA reductase (*MECR*), very-long-chain (3*R*)-3-hydroxyacyl-CoA dehydratase (*PHS1*) and palmitoyl-protein thioesterase (*PPT*), respectively (Table 5). In the comparison group HAvsHL, there were 10 up-regulated unigenes annotated as *KCS*, 1 up-regulated unigene annotated as *MECR* and 1 down-regulated unigene annotated as *PPT*. And in the comparison group HFvsHL, there were only 4 down-regulated unigenes, annotated as *KCS* and *PPT*. *KCS* and *PHS1* promote the formation of long-chain fatty acids from Malonyl-CoA. *MECR* and PPT promote the formation of palmitic acid from Acetyl-CoA. The results clearly show that high light promotes fatty acid elongation, especially through an increase in the expression of *KCS* and that acetate addition further increases the expression of *KCS*, whereas Fe^2+^ addition inhibited the expression of *KCS* to a certain extent. 

#### 2.4.3. The Effects on Photosynthesis-Antenna Proteins Pathway

**Figure 9:**
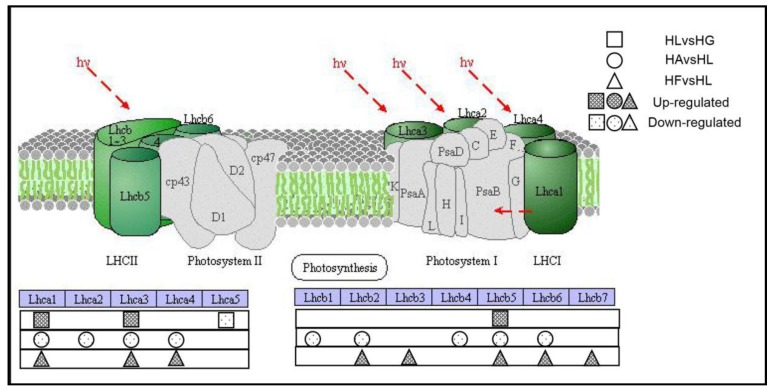
The pathway for photosynthesis-antenna proteins. *hv*: light energy; *Lhca1*–5: the genes encoding light-harvesting complex I chlorophyll a/b binding proteins 1–5; *Lhcb1*–7: encoding light-harvesting complex II chlorophyll a/b binding proteins 1–7.

The antenna proteins that exist in light-harvesting chlorophyll protein complexes in green plants act as peripheral antenna systems, enabling more efficient absorption of light energy. The DEGs related to the photosynthetic—antenna protein pathway (pathway map: ko00196) is shown in Table 6. The genes encoding light-harvesting complex I chlorophyll a/b binding proteins 1–5 (*LHCA1*–5) encode binding proteins in light-harvesting complex I and those encoding light-harvesting complex II chlorophyll a/b binding proteins 1–7 (*LHCB1*–7) encode binding proteins in light-harvesting complex II. In HLvsHG, the significantly up-regulated genes were *LHCA1*, *LHCA3* and *LHCB5*. More up-regulated genes were found in HFvsHL, including *LHCA1*, *LHCA3*, *LHCA4*, *LHCB2*, *LHCB3*, *LHCB5*, *LHCB6* and *LHCB7*. But in HAvsHL, all of the genes related to photosynthesis-antenna proteins were down-regulated (Figure 9). These results indicate that under high light condition, the genes related to photosynthesis-antenna proteins are partially up-regulated. Adding Fe^2+^ to cultures under high light condition up-regulated the expression of additional photosynthesis-antenna genes, whereas acetate down-regulated the expression of photosynthesis-antenna genes.

### 2.5. Analysis of the Results of Real-Time Quantitative PCR

**Figure 10:**
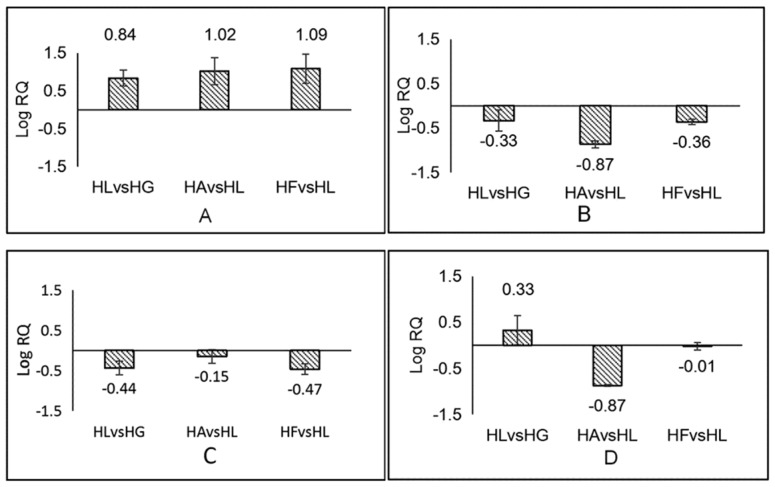
The results of qRT-PCR. “RQ” represented 2^−^^ΔΔ*C*t^ and “Log (RQ) > 0” means the gene was up-regulated, “Log (RQ) < 0” means the gene was down-regulated. (**A**–**D**) represented *crtZ*, *crtISO*, *lcyB* and *ZEP*, respectively.

To validate the sequencing results obtained by RNA-seq, four genes related to the carotenoid pathway were chosen for real-time PCR: *crtZ*, *lcyB*, *crtISO* and *ZEP*. The results of qRT-PCR showed that *crtZ* was significantly up-regulated in three experimental groups (HL, HA and HF) relative to the control group (HG). The level increased more than 10 fold in HAvsHL and HFvsHL and 6 fold in HLvsHG. *CrtISO* and *LcyB* were down-regulated in three experimental groups, decreasing 1.5–7.5 fold. *ZEP* was significantly down-regulated in the HAvsHG group (7.5 fold) (Figure 10). Overall, the expression levels of the four genes were similar in the results of RNA-seq and qRT-PCR. There were also some small differences between the two measurements, such as the up-regulated expression of *ZEP* in HLvsHG. This outcome might have been affected by different measurement methods. 

## 3. Discussion

In recent years, transcriptome analysis has become a hot tool for studying the level of gene transcription [19]. It has been reported in another transcriptome analysis of *H. pluvialis* that in the case of three genes—*crtZ*, *ZDS*, *PDS*—involved in the carotenoid biosynthesis pathway, *crtZ* was up-regulated after induction with both salicylic acid and jasmonic acid [9]. Using transcriptome analysis, Gwak found that beta-carotene hydroxylase (*BKT* or *crtZ*), phytoene synthase (*PSY*) and phytoene desaturase (*PDS*) were all up-regulated under high irradiance [7]. In our research, the expression of *crtZ* was affected by high light and was significantly promoted by acetate. It can be seen that β-carotene hydroxylase (*crtZ*) played a fundamental role in the astaxanthin biosynthesis pathway and many factors can promote its up-regulated expression, such as high light, acetate and specific hormones. High light impacted the expression of more genes involved in the carotenoid biosynthesis pathway, including *PDS*, *crtISO*, *LcyB*, *LUT1*, *LUT5* and *ZEP*, than the other two induction conditions, which shows that high light is the main driver for changes in expression of genes related to carotenoid biosynthesis. Acetate further promotes astaxanthin biosynthesis by enhancing the expression of *crtZ* and inhibiting the expression of *LcyE*. With regard to lipids, using a comparison of lipidomes and transcriptomes, Gwak found that synthesis of the storage lipid, triacylglycerol (TAG), was substantially stimulated under high irradiance and that it was regulated by expression of de novo fatty acid biosynthesis at the gene level [7]. Here, the genes involved in fatty acid related pathways, especially the fatty acid elongation pathway, were significantly affected by high light conditions. The addition of acetate further enhanced expression of the related genes (*KCS*, *MECR*). From all of the above, it can be seen that there was a similar effect of high light and acetate on the expression of genes involved in the carotenoid pathway and fatty acid pathway. There may be some link between the two processes where affecting one also affects the other. As for Fe^2+^, Kobayashi pointed out that Fe^2+^ promoted carotenoid formation by providing oxidative stress in acetate-induced cyst cells (through the method of inhibiting by potassium iodide) and the effect of Fe^2+^ could be substituted by active oxygen species [13]. Here, the genes most significantly affected by Fe^2+^ were related to photosynthesis, especially the photosynthesis - antenna proteins. The effect of Fe^2+^ on *H. pluvialis* was not directly on the genes involved in astaxanthin biosynthesis but rather indirectly by promoting oxidative stress, affecting the genes related to photosynthesis and thereby promoting astaxanthin accumulation.

## 4. Materials and Methods

### 4.1. Cell Culture and Treatments

*H. pluvialis* HOUC9 was stored in the Laboratory of Phycology at the Ocean University of China. 1000 mL conical flasks were used to culture 600 mL of algal cells in modified BBM (Bold’s Basal Medium) medium, with a 12 h:12 h light/dark cycle at 22 °C. The light intensity used for the green growth period was 30 μEm^−2^·s^−1^. When the cell concentration reached to (4–5) × 10^5^ cells/mL, induction treatments were initiated. 

The inductions were performed in 250 mL conical flasks with 50 mL inducing medium added to 50 mL of algal culture. The induction medium contained either acetate or Fe^2+^ as inducing salt. The experiments are grouped as follows: group HL was cultured under high light intensity (195 μEm^−2^·s^−1^) without any addition of inducing salt; group HA had acetate added to a total concentration of 2 g/L under the same high light intensity as group HL; group HF had Fe^2+^ added to a total concentration of 20 μM under the same high light intensity as group HL; group HG was the control group grown under normal light intensity (30 μEm^−2^·s^−1^) without addition of an inducing salt. Each group was comprised of three biological repeats and were cultured for 48 h. The algal cells were then collected and frozen in liquid nitrogen for RNA extraction. 

### 4.2. RNA Extraction and cDNA Library Construction

Total RNA of all samples was extracted using the EasyPure^®^ Plant RNA Kit (TransGen, Beijing, China). Then, four different methods were used to detect the quality and quantity of the total RNA. First, agarose gel electrophoresis was used to determine the RNA content. Second, a Nanodrop (Thermo Fisher Scientific, Waltham, MA, USA) spectrophotometer OD260/280 ratio, was used to measure its purity. Then, the concentration of the RNA was quantified using a Qubit fluorimeter (Thermo Fisher Scientific, Waltham, MA, USA). Finally, an Agilent 2100 bioanalyzer (Agilent, Beijing, China) was used to determine the integrity of the RNA accurately.

After the samples were characterized, the eukaryotic mRNA was enriched using Oligo(dT) magnetic beads. The mRNA was cut into short fragments using fragment Buffer and then used as the template for single-strand cDNA synthesis, after adding 6-bp random hexamers. The second-strand cDNA synthesis was performed by adding buffer, dNTPs, DNA polymerase I and RNase H. After purification of the double-stranded cDNA using AMPure XP beads, the ends were repaired and poly (A) was linked to the 3′-cDNA. Sequencing adapters, which contained a recognition site, were added to the end of the poly (A) sequence. After amplifying the double-stranded cDNA, the PCR amplification product was purified by AMPure XP beads. Finally, the cDNA library was constructed and the preliminary initial quantitation of the library was performed by Qubit 2.0 (Thermo Fisher Scientific, Waltham, MA, USA). Then, the cDNA concentration was diluted to 1.5 ng/µL. Subsequently, the insert size was checked with an Agilent 2100. After ascertaining that the insert size met the requirement, the effective concentration of the library was accurately quantified by Q-PCR (Effective concentration of library >2 nM) to guarantee the quality of the library. After detection of the libraries of all samples, the different libraries were pooled according to the effective concentration and the required amount of data and then sequencing was performed by Illumina HiSeq™ (Illumina, Santiago, CA, USA).

### 4.3. Quality Controls, De Novo Assembly and Clustering

The raw data were obtained and the base quality was tested using Q_phred_ software. If the sequence error rate was expressed in e, the base-quality value of Illumina HiSeq™ was expressed in Q_phred_, then the Q_phred_ = −10Log_10_ (e). And the distribution of the sequence error rate can reflect the quality of the sequenced data. Q10, Q20, Q30, Q40 represent an accuracy of 90%, 99%, 99.9%, 99.99%, respectively. The low-quality reads were filtered out, (i.e., reads containing primer/adaptor sequences, reads in which the proportion of undetermined bases were greater than 10%, reads with more than 50% proportion of bases with Q_phred_ ≤ 20 and so on) and the remaining clean reads were retained [20]. 

For transcriptome without reference sequence, the clean reads were de novo assembled by using Trinity [21] to obtain a reference sequence for subsequent analysis. Trinity combined three independent software modules (Inchworm, Chrysalis and Butterfly) to deal with a large amount of RNA-seq data successively. Inchworm was used to form contigs by decomposing reads, constructing k-mer (*k* = 25) dictionary, selecting seed k-mer and extending on both sides. Chrysalis was used to form components through the cluster of overlapped contigs and every component became a collection of possible characterization of variable shear isoform or homologous genes. And each component had the corresponding de Bruijn graph. Butterfly was used to output full-length transcripts of variable shear isoforms and comb transcripts that corresponded to paralogous genes through simplifying each component’s de Bruijn graph. After these three steps, the assembled results of TRINITY.fasta were finally obtained.

Then Corset was used to hierarchically cluster transcripts by expression patterns and the number of reads which had been blasted to transcripts [22]. After hierarchical clustering by Corset, the longest Cluster sequence was used for further analysis (Corset website: https://code.google.com/p/corset-project/).

### 4.4. Gene Function Annotation and Classification

Gene functional annotation was performed using seven databases, including Nr, Nt, Pfam, KOG/COG, SwissProt, KEGG and GO. By using Blast to search the Nr database, we could find functional information for genes of *H. pluvialis* and the similarity of gene sequences between *H. pluvialis* and related species. By GO annotation, the annotated genes were classified to the next terms of the three GO categories (BP biological process, CC cellular component, MF molecular function). By KO annotation, the annotated genes could be classified according to their KEGG metabolic pathway.

### 4.5. Analysis of Differentially Expressed Genes (DEGs)

For biologically repeated samples, DESeq [23] was used to do the differential analysis based on the negative binomial distribution. Using padj < 0.05 (Padj was the corrected value of *p*-value) as threshold value to screen out DEGs in different comparison groups, which had been set to 6 groups: HLvsHG, HAvsHG, HAvsHL, HFvsHG, HFvsHL and HAvsHF. Then two comparison combinations of the Venn diagram: HLvsHG-HAvsHG-HFvsHG and HAvsHL-HFvsHL, were set to show the number of total genes and specific genes between those comparison groups. As for the expression level of DEGs, cluster analysis was used to determine the expression patterns of DEGs under different experimental conditions based on the FPKM (expected number of Fragments Per Kilobase of transcript sequence per Millions base pairs sequenced) values. It is the most common method of gene expression level estimation [24]. Then the DEG enrichment analysis using GO and KEGG was used to identify gene function and metabolic pathway in the microalgae under different conditions. The Gene Ontology (GO) database (http://www.geneontology.org/) was used to map the DEGs to the term types and calculate the number of every term type. With the termed GOseq, the significantly enriched terms were found by comparing them to the whole genome background [25]. KEGG is the main public database for pathway determination [26]. KEGG enrichment could identify the principal metabolic pathways and signal transduction pathways of the DEGs. The KEGG significant enrichment was analyzed using a hypergeometric test to look for significantly enriched pathways of DEGs, compared to the all annotated genes by using KEGG Pathway as a unit. Computational formula of the analysis is:

$$p=1-\overset{m-1}{\underset{i=0}{\sum }}\frac{\left(\begin{array}{c} M\\ i \end{array}\right)\left(\begin{array}{c} N-M\\ n-i \end{array}\right)}{\left(\begin{array}{c} N\\ n \end{array}\right)}$$

N stands for the number of genes that can be annotated by pathways and *n* is the number of DEGs in N. M is the number of genes annotated to a certain pathway among all the genes and m is the number of DEGs in M. Those pathways with FDR (false discovery rate) ≤ 0.05 were defined as the significant enrichment pathways for DEGs. In this paper, FDR was a corrected *p*-value obtained by the BH method using KOBAS (2.0) [27].

### 4.6. DEG Validation and Expression Analysis

Based on the transcriptome result, genes related to carotenoid pathway (*crtZ*, *crtISO*, *lcyB*, *ZEP*) were chosen for a validity check of the transcriptome result, using real-time fluorescence quantitative PCR (qRT-PCR). The sample was prepared together with the transcriptome experiment and was kept in a −80 °C refrigerator for RNA extraction. The RNA was extracted using RNA kits (Takara) and the synthesis of cDNA was performed using a PrimeScript II 1st Strand cDNA Synthesis Kit (TaKaRa, Tokyo, Japan). Oligo dT primer and random 6-mer primers were both used as RT-primers [18].

The qRT-PCR was performed using a SYBR Premix Ex Taq™ II Kit (TaKaRa, Tokyo, Japan) with a 20 μL reaction system containing 10 μL 2× SYBR green Mastermix (TaKaRa, Tokyo, Japan), 1 μL each of the forward and reverse primers, 1 μL cDNA and 7 μL ddH_2_O. Reaction steps followed the program: 95 °C for 20 s, followed by 40 cycles of 95 °C for 15 s, 51 °C for 15 s, 72 °C for 20 s and reading the fluorescence signal, followed by 1 cycle of 95 °C for 15 s, 60 °C for 60 s, 95 °C for 15 s. Each qRT-PCR reaction was performed on 3 biological replicates. Ribulose-1,5-bisphosphate carboxylase/oxygenase (*Rbcl*: a house-keeping gene) was used as the reference gene. All primers were designed using the Primer Premier 5.0 software (Premier, Toronto, ON, Canada) and are listed in Table 7. The relative transcript level was calculated using the 2^−^^ΔΔ*C*t^ (RQ) method. Then a log function (logRQ) was used to analyze the qRT-PCR results. 

## 5. Conclusions

Based on the DEGs involved in carotenoid metabolism, the expression pattern of 9 genes *(PDS*, *crtISO*, *LUT1*, *LUT5*, *lcyB*, *lcyE*, *crtZ*, *CCD8*, *ZEP*) are different under different induction conditions. The results indicated that acetate and Fe^2+^ have different mechanisms for inducing astaxanthin production in *H. pluvialis*. High light is the direct factor for the changes in gene expression during the induction period. Acetate could further enhance expression of the genes involved in carotenoid and fatty acid synthesis. Fe^2+^ mainly influences the genes involved in photosynthesis and especially promoted the expression of genes related to photosystem I and photosystem II. Taken together, these results obtained through application of RNA-seq provide a foundation and orientation for future studies on *H. pluvialis*. 

## Figures and Tables

**Table 1:** List of data output quality.

Sample	Raw Reads	Clean Reads	Clean Bases	Error (%)	Q20 (%)	GC (%)
HG_1	59,081,916	57,542,600	8.63G	0.02	95.53	60.11
HG_2	53,033,442	51,513,440	7.73G	0.02	95.38	60.46
HG_3	63,249,144	61,459,678	9.22G	0.02	95.38	60.15
HL_1	55,314,470	53,253,670	7.99G	0.02	95.32	59.93
HL_2	56,631,072	55,112,470	8.27G	0.02	96.78	60.42
HL_3	50,409,762	49,054,352	7.36G	0.02	96.97	60.35
HA_1	60,762,278	58,220,746	8.73G	0.02	95.09	60.99
HA_2	54,539,640	51,357,156	7.7G	0.02	95.89	60.78
HA_3	65,324,354	62,001,576	9.3G	0.02	96.1	60.86
HF_1	58,086,534	55,405,818	8.31G	0.02	95.7	60.08
HF_2	68,968,618	65,327,172	9.8G	0.02	95.81	59.96
HF_3	66,261,124	63,319,584	9.5G	0.02	95.45	60.15

**Table 2:** Summary of the function annotation results for *H. pluvialis* unigenes in public protein databases.

Annotated Databases	Number of Genes	Percentage (%)
Annotated in NR	55,835	35.56
Annotated in NT	13,504	8.6
Annotated in KO	23,632	15.05
Annotated in SwissProt	36,522	23.26
Annotated in PFAM	58,643	37.35
Annotated in GO	60,846	38.75
Annotated in KOG	21,569	13.73
Annotated in all Databases	5918	3.76
Annotated in at least one Database	76,744	48.88
Total Unigenes	156,992	100

NR is NCBI non-redundant protein sequences database, NT is NCBI nucleotide sequences database, KO is KEGG Ortholog database, SwissProt is a manually annotated and reviewed protein sequence database, PFAM is Protein family database, KOG is Clusters of Orthologous Groups of proteins database and GO is Gene Ontology database.

**Table 3:** Number of DEGs (differentially expressed genes) in different comparison groups.

Comparison Groups	The Number of DEGs	The Number of Up-Regulated DEGs	The Number of Down-Regulated DEGs
HLvsHG	17,089	8243	8846
HAvsHG	15,555	7699	7856
HAvsHL	1824	1121	703
HFvsHG	21,271	10,496	10,775
HFvsHL	1900	1182	718
HAvsHF	4864	2844	2020

**Table 4:** Up-and-down regulated genes related to the carotenoid biosynthesis pathway.

KEGG Orthology Category Description	HLvsHG	HAvsHL	HFvsHL
*PDS*, *crtP*; 15-*cis*-phytoene desaturase [EC:1.3.5.5]	down	/	/
*crtISO*, *crtH*; prolycopene isomerase [EC:5.2.1.13]	down	/	/
*lcyB*, *crtL1*, *crtY*; lycopene β-cyclase [EC:5.5.1.19]	down	/	/
*lcyE*, *crtL2*; lycopene epsilon-cyclase [EC:5.5.1.18]	/	down	/
*LUT5*, *CYP97A3*; β-ring hydroxylase [EC:1.14.-.-]	up	/	/
*LUT1*, *CYP97C1*; carotene epsilon-monooxygenase [EC:1.14.99.45]	up	/	/
*crtZ*; β-carotene 3-hydroxylase [EC:1.14.13.129]	ns	up	/
*CCD8*; carlactone synthase/all-trans-10′-apo-β-carotenal 13,14-cleaving dioxygenase [EC:1.13.11.69 1.13.11.70]	up	up	/
*ZEP*, *ABA1*; zeaxanthin epoxidase [EC:1.14.15.21]	down	/	/

“Up” means all of the DEGs were up-regulated, “down” means all of the DEGs were down-regulated, “ns” means the no significant change, with the DEGs both up-regulated and down-regulated, “/” means no DEGs.

**Table 5:** The up-and-down regulated genes related to the fatty acid elongation pathways.

KEGG Orthology Category Description	HLvsHG	HAvsHL	HFvsHL
*KCS*; 3-ketoacyl-CoA synthase [EC:2.3.1.199]	up	up	down
*PHS1*, *PAS2*; very-long-chain (3*R*)-3-hydroxyacyl-CoA dehydratase [EC:4.2.1.134]	up	/	/
*MECR*, *NRBF1*; mitochondrial *trans*-2-enoyl-CoA reductase [EC:1.3.1.38]	up	up	/
*PPT*; palmitoyl-protein thioesterase [EC:3.1.2.22]	up	down	down

“Up” means all of the DEGs were up-regulated, “down” means all of the DEGs were down-regulated, “/” means no DEGs.

**Table 6:** The up-and-down regulated genes related to photosynthetic—antenna protein pathway.

KEGG Orthology Category Description	HLvsHG	HFvsHL	HAvsHL
*LHCA1*; light-harvesting complex I chlorophyll a/b binding protein 1	up	up	down
*LHCA2*; light-harvesting complex I chlorophyll a/b binding protein 2	ns	ns	down
*LHCA3*; light-harvesting complex I chlorophyll a/b binding protein 3	up	up	down
*LHCA4*; light-harvesting complex I chlorophyll a/b binding protein 4	ns	up	down
*LHCA5*; light-harvesting complex I chlorophyll a/b binding protein 5	down	/	/
*LHCB1*; light-harvesting complex II chlorophyll a/b binding protein 1	ns	ns	down
*LHCB2*; light-harvesting complex II chlorophyll a/b binding protein 2	ns	up	down
*LHCB3*; light-harvesting complex II chlorophyll a/b binding protein 3	/	up	/
*LHCB4*; light-harvesting complex II chlorophyll a/b binding protein 4	ns	ns	down
*LHCB5*; light-harvesting complex II chlorophyll a/b binding protein 5	up	up	down
*LHCB6*; light-harvesting complex II chlorophyll a/b binding protein 6	/	up	down
*LHCB7*; light-harvesting complex II chlorophyll a/b binding protein 7	/	up	/

“Up” means all of the DEGs were up-regulated, “down” means all of the DEGs were down-regulated, “ns” means no significant change, with the DEGs both up-regulated and down-regulated, “/” means no DEGs.

**Table 7:** Primers used for qRT-PCR.

Gene Name	Primers
*Rbcl* (ribulose-1,5-bisphosphate carboxylase/oxygenase: Genbank-KR914695.1)	*Rbcl1*: ACGAATGTTTACGCGGTGGTCT
	*Rbcl2*: GGTACACCCAACTCCTTAGCA
*CrtZ* (carotenoid hydroxylase: Genbank-KP866868.1)	*CrtZ1*: ATCTTCGCCACCTACCTGAG
	*CrtZ2*: CGGGCAGTCCATTGATGATT
*LcyB* (lycopene beta cyclase: Genbank-AY182008.1)	*LcyB1*: CTTTGACCCAGCACGACTTG
	*LcyB2:* TTAGGAACTTCTCGCCCTCT
*CrtISO* (carotenoid isomerase: Genbank-KP780062.1)	*CrtISO1*: GCACCACTAACCTCATCACA
	*CrtISO2*: CCTTCCAGCAGTCGTCATAA
*ZEP* (zeaxanthin epoxidase: Genbank-KP780061.1)	*ZEP1*: TACCTCAACCCTGCTCCTGA
	*ZEP2*: TTCGCCCTTCTCAAGCCTGT

## Supplementary Materials

Supplementary materials can be found at www.mdpi.com/1422-0067/19/1/175/s1.
